# Molecular and phenotypic profiles of carbapenem- and third-generation cephalosporin-resistant *Klebsiella pneumoniae* isolates from the fecal microbiota of pediatric patients with COVID-19

**DOI:** 10.1186/s12866-026-05426-5

**Published:** 2026-07-23

**Authors:** Mahmoud A.F. Khalil, Mahmoud R. M. El-Ansary, Sara I. AboElnour, Shaimaa Madkour, Walid F. Elkhatib, Eman E. Mahmoud, Eman Fares, Fatma A. Ahmed

**Affiliations:** 1 Department of Microbiology and Immunology, Faculty of Pharmacy, Fayoum University, Fayoum, 63514 Egypt; 2 Department of Medical Microbiology and Immunology, Faculty of Medicine, Misr University for Science and Technology (MUST), Giza, 12566 Egypt; 3 Department of Pediatrics, Faculty of Medicine, Fayoum University, Fayoum, Egypt; 4 Department of Microbiology & Immunology, Faculty of Pharmacy, Galala University, New Galala city, Suez, Egypt; 5 Microbiology and Immunology Department, Faculty of Pharmacy, Ain Shams University, African Union Organization St., Abbassia, Abbassia, Cairo 11566 Egypt; 6 Department of Clinical and Chemical Pathology, Faculty of Medicine, Fayoum University, Fayoum, 63514 Egypt; 7 Department of Endemic Medicine, Faculty of Medicine, Fayoum University, Fayoum, Egypt; 8 Medical Microbiology and Immunology Department, Faculty of Medicine, Fayoum University, Fayoum, Egypt

**Keywords:** *Klebsiella pneumoniae*, Carbapenem resistance, ESBL, Gut colonization, Pediatric COVID-19

## Abstract

**Background:**

Gut colonization with multidrug-resistant (MDR) *Klebsiella pneumoniae* is commonly associated with an increased risk of extraintestinal infections among hospitalized patients. Extensive antibiotic exposure during the COVID-19 pandemic, is a critical contributing factor for the emergence of resistant strains. This investigation sought to elucidate the phenotypic and molecular resistance traits of carbapenem-resistant *K. pneumoniae* (CRKP) and extended-spectrum β-lactamase–producing *K. pneumoniae* (ESBL-KP) colonizing the gut of pediatric patients with confirmed COVID-19.

**Methods:**

A cross-sectional study was conducted from October 2020 to March 2022 at a tertiary pediatric hospital in Fayoum, Egypt. Fecal samples were collected from hospitalized pediatric COVID-19 patients. *K. pneumoniae* isolates were identified using standard microbiological methods. Antimicrobial susceptibility testing was carried out in accordance with the CLSI guidelines. ESBL and carbapenemase production were assessed phenotypically, while resistance genes were detected by multiplex and uniplex PCR. Microtiter plate method was used to assess biofilm formation.

**Results:**

*K. pneumoniae* was detected in 36 of 71 patients (50.7%) using antibiotic-supplemented selective culture. The isolated strains were exclusively resistant, including 22 CRKP and 14 ESBL-KP. Colistin susceptibility was maintained in all isolates. CRKP strains showed significantly increased rate of resistance to fluoroquinolones and amikacin compared with ESBL-KP. *Bla*_NDM_ gene (54.5%) and *bla*_KPC_ (45.5%) were detected among CRKP isolates. ESBL-associated genes *bla*_CTX-M_, *bla*_TEM_, and *bla*_SHV_ were prevalent in both groups. Biofilm formation was common and comparable between CRKP and ESBL-KP isolates.

**Conclusion:**

A high prevalence of fecal carriage of MDR *K. pneumoniae*, driven by carbapenemase-producing strains, was observed among hospitalized pediatric COVID-19 patients. These outcomes highlight the necessity for routine colonization surveillance, optimized antimicrobial stewardship, and strengthened infection control strategies in pediatric settings.

**Supplementary Information:**

The online version contains supplementary material available at 10.1186/s12866-026-05426-5.

## Background

*K. pneumoniae* is a Gram-negative bacterium commonly found in the human intestinal tract. It is the main cause of many extra intestinal infections that are greatly non susceptible to last-line antibiotics [1]. Newborns, infants, and children with compromised immune response are common susceptible host for serious infections with *K. pneumoniae* [2, 3]. During the last decades *K. pneumoniae* exhibited a marked ability to acquire and develop variable resistance mechanisms as a superbug. Horizontal gene transfer occurs between different strains and species in the intestinal microbiota. The mechanisms involved are commonly encoded on mobile genetic elements such as plasmids and integrons [4–6]. The gut colonization with MDR *K. pneumoniae*, serves as a reservoir for resistance. Bacteria in feces can cause endogenous infections or disseminate within medical facilities. It raises the chances of severe infections, such as, pneumonia, bloodstream infections and surgical site infections [7, 8]. Colonization is a critical finding, as it is an essential epidemiological trigger of AMR, especially in healthcare settings where infection control practices may be applied incompletely [9, 10]. Colonization with MDR *K. pneumoniae* is usually associated with clinical severity and prolonged antibiotic use [11]. Many broad spectrum antibiotics such as third-generation cephalosporins, carbapenems, aminoglycosides, and fluoroquinolones induce the selection and persistence of MDR *K. pneumoniae* [12]. The development of resistance is significantly correlated with exposure to broad spectrum antibiotics [13, 14].

Disruptions of the microbial balance caused by antibiotics may last for several weeks or potentially months following the antibiotic therapy. The effect could be marked and long-lasting in newborns and young children, whose gut bacteria are still maturing, which could affect their microbial resistance characteristics for many years in the future [15, 16]. Moreover, the severity of the clinical state has a vital impact in microbial colonization dynamics. For instance patients under mechanical ventilation are more susceptible to breaches in mucosal barriers, enhance the microbial translocation from the gut to sterile sites. In addition, a serious health issue is often linked to systemic inflammation, changes in intestinal permeability, and alterations in the immune response [17, 18].

CTX-M-15, TEM, and SHV are the most prevalent ESBL enzymes, whereas *K. pneumoniae* carbapenemase (KPC), New Delhi metallo-β-lactamase (NDM), oxacillinase-48 (OXA-48), and Verona integron-encoded metallo-β-lactamase (VIM) are the primary causes of carbapenem resistance [19]. Several outbreaks in neonatal and pediatric healthcare units have been linked to hypervirulent MDR *K. pneumoniae* clones as ST258, ST15, and ST11 [20, 21]. Despite the increasing challenge of MDR *K. pneumoniae*, routine surveillance of fecal carriage in pediatric patients still uncommon, especially in resource-limited settings. Without baseline data on bacterial colonization rates and the prevalence of antimicrobial resistance genes, infection prevention and control teams lack the evidence needed to develop effective strategies to limit transmission within healthcare facilities [22, 23].

Additionally, the COVID-19 pandemic has further intensified the existence risk. While there is no direct connection between *K. pneumoniae* and SARS-CoV-2, the overuse of empirical antibiotic therapy during pandemic contributed to these challenges. Many hospitalized COVID-19 patients were exposed to broad-spectrum antibiotics as a precaution against bacterial co-infection. Pandemic-related strain on healthcare systems, including compromised infection control measures and increased patient loads, likely accelerated the transmission of MDR pathogens such as *K*. *pneumoniae* [24, 25]. Reports on fecal colonization of MDR *K. pneumoniae* in pediatric patients remains limited, particularly regarding its relationship with clinical outcomes and treatment patterns. Few studies have explored the concordance between genotypic resistance (presence of resistance genes) and phenotypic resistance (antibiotic susceptibility) in colonizing *K. pneumoniae* strains a topic with profound implications for surveillance and empirical treatment [12, 26].

In the light of this gaps, there is an urgent need for systemic investigation to report the dissemination, resistance mechanisms, and clinical implications of fecal carriage of MDR *K. pneumoniae* among pediatric. Characterization of risk factors, identifying the antibiotic effect, and studying correlations with clinical severity are essential for improving antimicrobial stewardship, strengthening infection control, and mitigating the escalating burden of antimicrobial resistance [27–29]. Current study aims is to analyze the frequency and resistance profiles of MDR *K. pneumoniae* isolated from fecal samples from hospitalized pediatric patients, and analyzing the relationship between colonization, antibiotic exposure, and clinical outcomes.

## Materials and methods

### Study design and setting

This current work was a cross-sectional observational analysis conducted at [Fayoum University Pediatrics Hospital], a tertiary care pediatric hospital [pediatric intensive care (PICU)] in [Fayoum, Egypt], from [October 2020] to [March 2022]. The main target of this study was to investigate the prevalence of fecal carriage of multidrug-resistant (MDR) *K. pneumoniae* in hospitalized pediatric patients and to analyze its relation with clinical implications and used antibiotic therapy.

### Clinical data collection

Clinical and demographic data were collected using a standardized data extraction sheet. Variables included: Demographics: Age, sex; Clinical severity, Antibiotic Exposure Assessment. All antibiotic exposures were recorded within the timeframe from admission to the point of fecal sample collection. Fecal samples were collected one week after completion the antibiotic course. Only hospitalized pediatric patients with laboratory confirmed COVID-19 via Reverse Transcriptase-Polymerase Chain Reaction (RT-PCR), who fulfilled the predefined eligibility criteria and had fecal samples available during the study period were included. The study was additionally limited by patient availability, consent acquisition, and microbiological processing capacity during the pandemic period. Other exclusion criteria included: antibiotic therapy within the past 60 days, prior hospitalization or previous hospital admission. According to national and institutional guidelines for pediatric COVID-19 care, the disease is classified into four severity levels (mild, moderate, severe, and critical categories). The presence of minor upper respiratory tract symptoms without signs of hypoxia or pneumonia is referred to as the mild form. The moderate type is characterized by oxygen saturation (SpO2) ≥ 94% on room air and signs of pneumonia without significant respiratory distress. Severe pneumonia, respiratory distress, oxygen saturation < 94%, or the requirement for oxygen therapy are signs of severe forms. Critical cases are reserved for the most dangerous outcomes ARDS, respiratory failure requiring mechanical ventilation, shock, pediatric multisystem inflammatory syndrome, and multiorgan dysfunction requiring intensive care [30].

### Sample collection and isolation of *K. pneumoniae*

Fecal specimens were obtained under aseptic conditions and directly transported to the microbiological analysis. Selection of Extended-Spectrum β-Lactamase (ESBL)-producing Gram-negative pathogen was performed using MacConkey agar containing cefotaxime (Sigma Aldrich, USA) 1 µg/ml [31]. Carbapenem-resistant Enterobacteriaceae (CRE) were screened using MacConkey agar supplemented with meropenem 1 µg/mL [32]. Presumptive *K. pneumoniae* colonies were identified by standard biochemical assays and confirmed using an automated identification system (VITEK 2). Single colonies were purified by streaking on macConkey agar plates.

### Antimicrobial susceptibility testing

Kirby–Bauer disk diffusion test was used to define the antimicrobial sensitivity patterns according to the guidelines of Clinical and Laboratory Standards Institute (CLSI) 2023. The tested β-lactams included ceftriaxone (30 µg), cefepime (30 µg), piperacillin–tazobactam (100/10 µg), ampicillin–sulbactam (10/10 µg), and amoxicillin–clavulanate (20/10 µg); meropenem (10 µg). For aminoglycosides amikacin (30 µg) and gentamicin (10 µg) was used; while, fluoroquinolones included ciprofloxacin (5 µg) and levofloxacin (5 µg) (HiMedia Laboratories Pvt. Ltd., Mumbai, India). The broth microdilution assay, was used to test the minimum inhibitory concentrations (MICs) for colistin (Sigma-Aldrich, St. Louis, MO, USA) and meropenem (AstraZeneca, Cambridge, UK). Cation-adjusted Mueller-Hinton broth (MHA) (BD, Sparks, MD, USA) were used to carry out the test [33]. Both Kirby–Bauer and broth microdilution were employed to cross-validate results and meet CLSI requirements for different drug categories. Quality control was ensured using *E. coli* ATCC 25,922, per CLSI guidelines.

### Phenotypic detection of Extended-Spectrum β-Lactamases (ESBLs)

ESBLs activity was detected phenotypically by using double disk synergy test (DDST) as previously described [34]. Briefly; bacterial isolates were inoculated into MHA plates and evaluated for synergetic interaction between amoxicillin/clavulanic acid (20 µg/10µg), with ceftazidime (30 µg/mL), cefotaxime (30 µg/mL), cefepime (30 µg/mL), and aztreonam (30 µg/mL) (HiMedia Laboratories Pvt. Ltd., Mumbai, India). All plates were incubated for 18–24 h at 37 ^ο^C [34] .

### Phenotypic detection of carbapenemases by Triton Hodge Test (THT)

Fifty microliter of Triton X-100 (Sigma-Aldrich, St. Louis, MO, USA) was dispensed onto the center of a MHA plate and uniformly distributed using sterile swabs. After absorption period (10 min), the Triton Hodge Test (THT) was carried out according to the Modified Hodge Test (MHT) protocol, as previously reported [35, 36]. In brief, a lawn culture of *E. coli* ATCC 25,922 was prepared on MHA, followed by locating of a meropenem disk (10 µg) (HiMedia Laboratories Pvt. Ltd., Mumbai, India) at the plate center. The examined isolate was streaked from the disk margin to the plate exterior. After incubation, carbapenemase activity was determined by the presence of a characteristic cloverleaf-shaped indentation indicating enhanced growth of the indicator strain toward the carbapenem disk. *K. pneumoniae* ATCC BAA-1705 and ATCC BAA-1706 were used as positive and negative controls, respectively.

### Crystal violet microtiter plate biofilm assay

The microtiter plate approach was used to assess the biofilm forming capacity of bacterial isolates as previously mentioned [37]. In brief, Luria-Bertani (LB) broth enriched with 1% glucose (180 µL) and 20 µL of bacterial culture **(**1 × 10⁶ CFU/mL**)** were placed in sterile 96 well microtiter plates. After static incubation at 37 °C for 24 h Wells were gently washed three times with sterile phosphate-buffered saline (PBS) to remove planktonic cells. The plates were then dried and stained with 1% crystal violet. The excess dye was removed and washed three successive times. Glacial acetic acid 33% (v/v), was used to solubilize the attached stain. The absorbance was checked at 570 nm (OD₅₇₀). The assay was carried out in triplicate, and mean absorbance values were estimated. The cutoff OD (ODc) was identified as the mean OD of the negative control plus three standard deviations, with biofilm formation categorized as strong (OD > 4×ODc), moderate (2×ODc < OD ≤ 4×ODc), weak (ODc < OD ≤ 2×ODc), or absent (OD ≤ ODc) [37]. Sterile broth without inoculation was used as a negative control, while *K. pneumoniae* ATCC 13,883 was used as a positive control.

### Molecular detection of resistance genes

DNA was extracted using the boiling method. A pure isolated colonies were suspended in sterile nuclease-free water, followed by boiling at 100 °C for 10 min and immediate cooling on ice. After centrifugation, the supernatant containing the extracted DNA was recovered and maintained at − 20 °C [38]. DNA concentration and purity were quantified by NanoDrop spectrophotometer, by measuring absorbance at 260/280 nm prior to PCR amplification. ESBLs genes were confirmed by using two multiplex PCR reactions. One multiplex assay involve *bla*TEM/ *bla* SHV/ *bla*OXA-1, while the other reaction included detection of *bla*_CTX−M_ (including phylogenetic groups 1, 2 and 9) [39]. On the other hand, one uniplex PCR was used for detection of *bla*_CTX−M−8/−25_. The PCR reaction conditions were as follows: initial denaturation at 95 °C for 5 min; 30 cycles of denaturation at 94 °C for 30s, annealing at 56 °C for 30s, and extension at 72 °C for 1 min; and a final extension at 72 °C for 10 min [39]. In addition, *bla*_KPC_ and metallo-β-lactamase (MBL) genes, *bla*_VIM_, *bla*_IMP_, and *bla*_NDM_ were investigated as previously described [40, 41]. Briefly, twenty microliter PCR reaction contained 1× PCR master mix, 0.2 µM of each primer (Table S1), and 2 µL of template DNA. The reaction parameters were adjusted as following: initial denaturation at 95 °C for 5 min; 30 cycles of 94 °C for 30 s, 52 °C for 40 s, and 72 °C for 1 min; and final extension at 72 °C for 10 min. PCR products were analyzed by agarose gel electrophoresis and visualized under UV illumination after ethidium bromide staining. Positive and negative controls were included in each run.

### Data analysis

Microsoft Excel and SPSS version 25.0 were used to record and evaluate the data. A statistically significant p-value was one that was less than 0.05.

## Results

### Demographic and clinical characteristics of patients

The current work included 71 pediatric patients with confirmed COVID 19. The studied *K. pneumoniae* isolates were collected from 50.7% (36/71) of the patients. The CRKP carriers, 22 in number, had a median age of 10.5 months of age (range: 1–18 months), with a predominance of females, 63.6%. The ESBL carriers, 14 in number, had a median age of 7.5 months of age (range: 2 months-3 years), with male predominance, 64.3%. The majority of the patients, 72.7% and 64.3% for CRKP and ESBL, respectively, had severe manifestations of COVID-19, predominantly pneumonia and septic shock. Common comorbid conditions included hydrocephalus (Table 1).

**Table 1:** Demographic and clinical characteristics of pediatric patients with crkp and esbl-producing *K. pneumoniae* colonization

	CRKP (*n* = 22)		ESBL-KP (*n* = 14)		
Characteristic	*n*	%	*n*	%	*p*-value
Patient Demographics					
Age, median [IQR], months	10.5 [4–12]	—	7.5 [2–12]	—	0.609
Age range, months	1–18	—	2–36	—	—
Age group		—		—	0.488
* <6 months*	8	36.4%	6	42.9%	—
* 6–12 months*	10	45.4%	6	42.9%	—
* 13–24 months*	4	18.2%	1	7.1%	—
* >24 months*	0	0.0%	1	7.1%	—
Sex		—		—	0.171
Male	8	36.4%	9	64.3%	—
Female	14	63.6%	5	35.7%	—
COVID-19 Disease Severity					
COVID-19 severity distribution		—		—	0.803
Mild	2	9.1%	2	14.3%	—
Moderate	4	18.2%	3	21.4%	—
Severe	13	59.1%	6	42.9%	—
Critical	3	13.6%	3	21.4%	—
Comorbidity and Complications					
Pneumonia	18	81.8%	12	85.7%	1.000
Septic shock	7	31.8%	3	21.4%	0.706
Hydrocephalus	7	31.8%	2	14.3%	0.431
Down syndrome	0	0.0%	2	14.3%	0.144
Gastroenteritis	1	4.5%	0	0.0%	1.000
Post-COVID cardiomyopathy	1	4.5%	0	0.0%	1.000
Intussusception	1	4.5%	0	0.0%	1.000

A *p-value* > 0.05 across all categories indicates that there are no statistically significant differences in demographics, COVID-19 severity, or primary comorbidities between the CRKP and ESBL groups, suggesting the cohorts are well-matched for comparative analysis

All the ESBL isolates were sensitive to meropenem. However, the CRKP isolates were resistant to meropenem (MIC range, 8–128 µg/mL). Colistin susceptibility was retained for all the isolates (MIC < 1 µg/mL). The CRKP isolates exhibited higher resistance rates to aminoglycosides and fluoroquinolones when compared with ESBL isolates (Table 2).

**Table 2:** Comparison of phenotypic antibiotic resistance between CRKP and ESBL-KP clinical isolates

#		CRKP (*n* = 22)		ESBL-KP (*n* = 14)		Statistical Comparison	
	Antibiotic	*n* Resistant	% Resistant	*n* Resistant	% Resistant	*p*-value	Sig.
*Aminoglycoside*							
1	Amikacin	17	77.3%	6	42.9%	0.082	ns
2	Gentamicin	17	77.3%	5	35.7%	0.032	*
*4th-gen Cephalosporin*							
3	Cefepime	22	100.0%	9	64.3%	0.005	**
*3rd-gen Cephalosporin*							
4	Ceftriaxone	22	100.0%	13	92.9%	0.389	ns
*Carbapenem*							
5	Meropenem	22	100.0%	0	0.0%	< 0.001	***
*β-Lactam/Inhibitor*							
6	Piperacillin-Tazobactam	22	100.0%	11	78.6%	0.051	ns
*Fluoroquinolone*							
7	Ciprofloxacin	19	86.4%	1	7.1%	< 0.001	***
8	Levofloxacin	19	86.4%	1	7.1%	< 0.001	***
*Polymyxin*							
9	Colistin	0	0.0%	0	0.0%	1.000	ns

Data are presented as *n* (%) resistant isolates. CRKP, *n* = 22; ESBL-KP, *n* = 14. *P-values* from Fisher’s exact test (expected cell count < 5) or chi-squared test. Significance: *p < 0.05 **p < 0.01 ***p < 0.001 ns = not significant

### Phenotypic detection of ESBL and carbapenemase production

The double-disc synergy test (DDST) was used to screen the extended-spectrum β-lactamase (ESBL) activity among *K. pneumoniae* isolates. Out of 14 isolates, 12 (85.7%) showed a synergistic interaction, confirming ESBL production, while 2 isolates (14.3%) were negative. Carbapenemase activity was investigated among CRKP isolates using the Triton Hodge Test (THT). Among the 22 isolates subjected to phenotypic testing, 20 (90.9%) showed positive carbapenemase activity (Figure S1), whereas 2 isolates (9.1%) were negative.

### Biofilm formation results

Biofilm production was common in both groups, with no significant difference (81.8% CRKP vs. 85.7% ESBL, *p* = 0.76). Most biofilm producers were classified as “mild” producers. There were significant differences in resistance gene profiles.

### Molecular detection of resistance genes

**Fig. 1:**
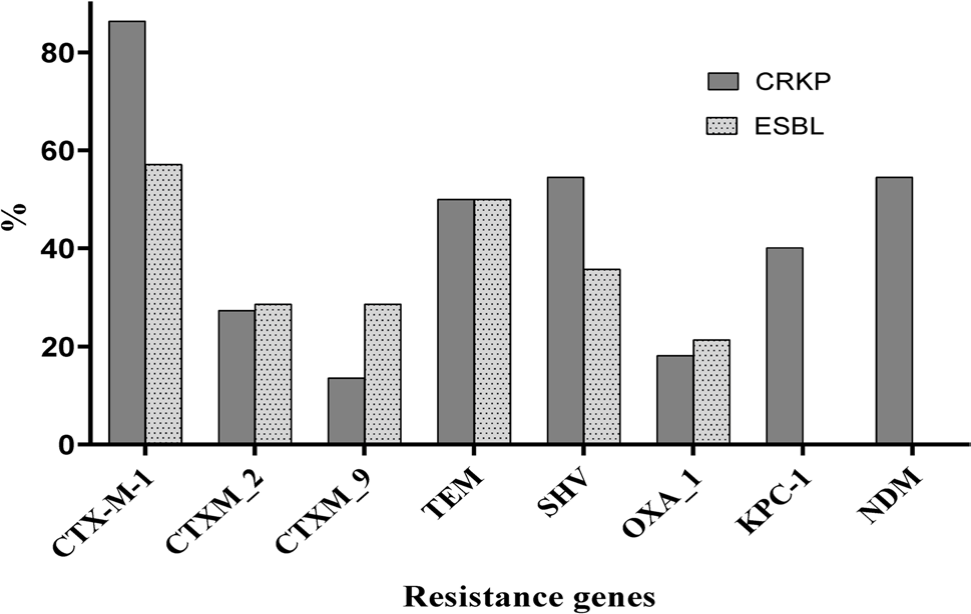
Comparative analysis of antimicrobial resistance gene distribution between CRKP and ESBL Isolates. The bar chart illustrates the percentage (%) of isolates harboring specific β-lactamase resistance genes. Gray bars indicate CRKP and dotted bars indicate ESBL. Notably, carbapenemase genes (KPC-1 and NDM) are detected mainly in the CRKP group

NDM was only identified among (12/22) CRKP isolates (54.5% vs. 0%, *p* < 0.0001), as was KPC (10/22) (45.5% vs. 0%, *p* < 0.0001). CTXM-1 was more common among CRKP (86.4% vs. 57.1%, *p* = 0.08). TEM and SHV had similar distributions between groups (50.0% vs. 50.0% and 54.5% vs. 35.7%, respectively) (Fig. 1) (Table S2).

### Antibiotic exposure patterns during hospitalization

**Fig. 2:**
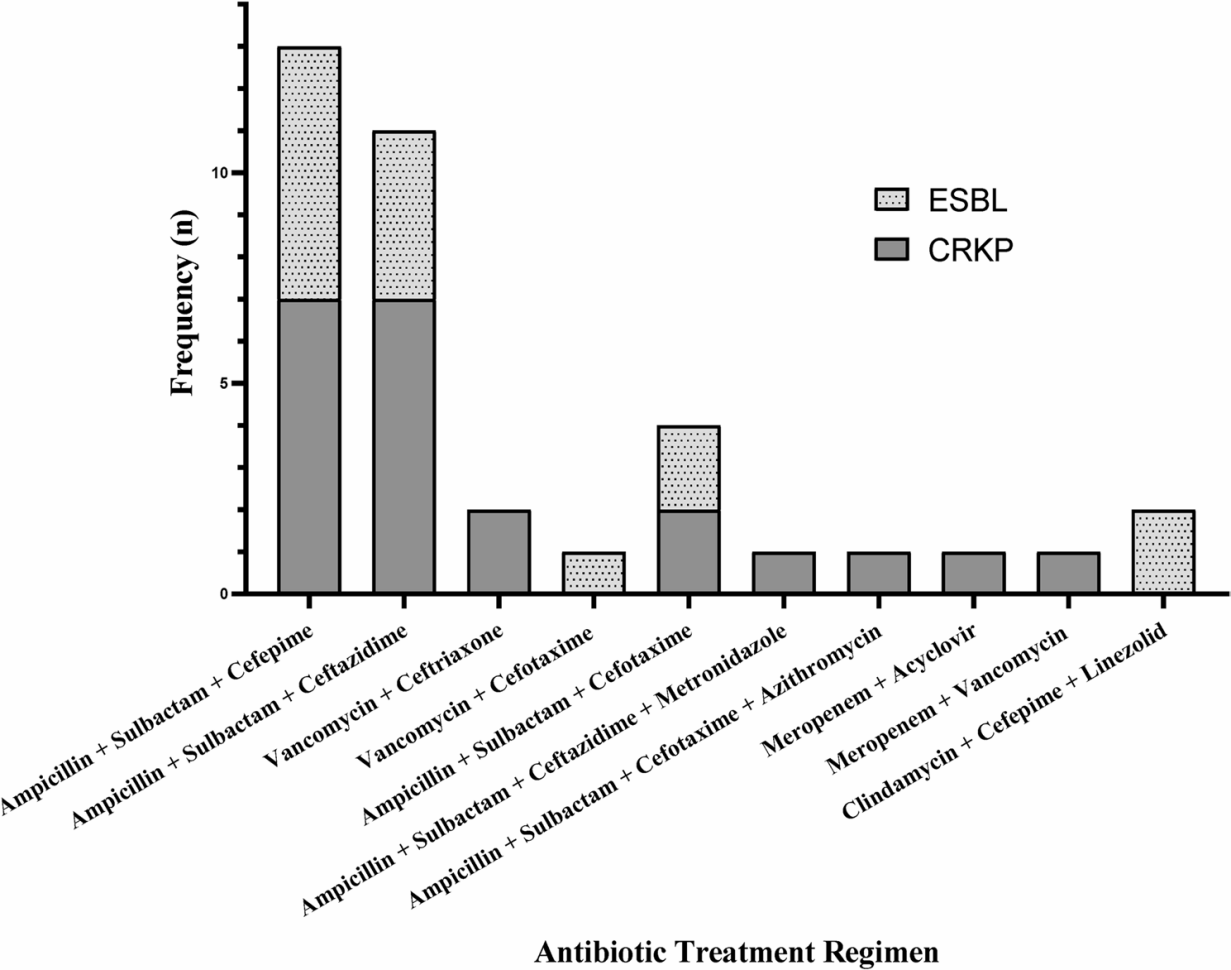
Distribution of antibiotic treatment regimens. The bar chart represents the total frequency (count) of specific antibiotic combinations or monotherapies used during clinical management. Gray bars indicate CRKP and dotted bars indicate ESBL

Antibiotic usage during hospitalization was reviewed and classified based on: Type of antibiotics administered included: carbapenem: meropenem, penicillin: ampicillin/sulbactam, cephalosporin: ceftazidime, cefepime, glycopeptide: vancomycin, macrolide: azithromycin, linezolid, clindamycin, and metronidazole. The top two combinations remain Ampicillin + Sulbactam + Cefepime and Ampicillin + Sulbactam + Ceftazidime, confirming that this dual β-lactam/cephalosporin strategy is the primary empirical choice (Fig. 2) (Table S3).

## Discussion

The development of MDR *K. pneumoniae* infections within the susceptible populations of hospitalized children is a great health concern. The current report provides important information about fecal colonization, resistance traits, and the demographic and clinical characteristics associated with MDR *K*. *pneumoniae* colonization among children who have been hospitalized due to COVID-19. Our findings demonstrated a worrying rise in colonization by CRKP and highlighted the potential jeopardy associated with antibiotic exposure–related stress. Our isolates were recovered from 36 out of 71 (50.7%) hospitalized pediatric patients, with a substantial part being resistant strains: CRKP (*n* = 22) and ESBL (*n* = 14). Colonization with MDR pathogens act as the leading cause for spreading of many invasive infections. This threat is particularly documented among susceptible pediatric patients, such as extremely depleted or immunocompromised children, who often encounter greater rates of colonization and consequent mortality [42–44].

Our data revealed no statistically significant differences between CRKP producers and ESBL producers when it came to median ages, COVID-19 degree of severity, as well as prevalent complications. Nevertheless, the current study indicates that the studied population is under considerable stress. The existence of severe underlying disorder, would considerably elevate the exposure rates to many classes of antibiotics. This consequently, enhance the vulnerability to colonization with highly-resistant pathogens, eventually creating a vicious cycle of resistance susceptibility [45–47].

However, assessing the sensitivity to several antibiotic classes revealed a significant difference of resistance findings and further validated the fact that all CRKP isolates (100%) were resistant to meropenem, which is one of the main therapeutic option in severe infections [48]. Through the current work, we selected meropenem as the representative carbapenem for several clinical and microbiological purpose. Meropenem was approved to have lower risk of seizures (0.5% vs. 1.5-3%), making it more appropriate for pediatric patients, with neurological involvement or renal dysfunctions [49–51]. Unlike ertapenem, meropenem showed broader spectrum of activity against most common nosocomial pathogens [52]. Additionally, meropenem is the CLSI recommended agent for screening carbapenem resistance in Enterobacterales, making it good choice for surveillance and epidemiological studies of antimicrobial resistance patterns [53].

Likewise, the significant aspect with regard to CRKP isolates is the low level of susceptibility to the key therapeutic alternative, such as fluoroquinolones (ciprofloxacin and levofloxacin, 86.4%), and high resistance rates to aminoglycosides as amikacin (77.3%), in regard to the sensitivity profiles of the ESBL strains. This comes in accordance with many reports recognized the cross resistance of CRKP to other antibiotics classes as fluoroquinolones and aminoglycosides [54–56]. Moreover, colistin susceptibility was retained for all the isolates (MIC < 1 µg/mL). Several reports highlighted the importance of colistin as essential therapeutic alternative for the management of CRKP infections [57, 58].

Phenotypic detection of ESBL and carbapenemase activity usually corresponded closely with the genetic outcomes. The Double Disk Synergy Test (DDST) observed ESBL production in 85.7% of the ESBL strains, while the Triton Hodge Test (THT) was positive in 90.9% of the CRKP group. The investigation of ESBL in *K. pneumoniae* using DDST assay is of increased sensitivity, up to 97.4% in comparison with chromogenic media. Across several clinical reports, DDST was able to identify ESBL production among 60–85% of molecular-positive isolates [59]. Two CRKP isolates were phenotypically negative for carbapenemase activity; this could be attributed to alternative resistance mechanisms such as AcrAB-TolC efflux pump overexpression, OmpK35/OmpK36 porin downregulation, or synergistic β-lactamase accumulation [60].

This work found a high rate of biofilm formation in both CRKP (81.8%) and ESBL (85.7%) isolates, with no significant difference between the groups. Biofilm production, even if classified as “mild” producers, is a significant virulence factor, help pathogen to combat host immunity and reduce the susceptibility to antibiotics. Many studies reported biofilm production among MDR *K. pneumoniae*. A Study by Di Domenico and coworkers showed the dissemination of strong biofilm producers among 55.8% CRKP isolates from colonized and infected patients [61]. On the other hand, weak biofilm production among the majority of ESBL- and KPC-producing isolates were recognized by Sabenca et al. [62]. Furthermore, no significant differences appear between CRKP and ESBL groups in multiple analyses, aligning with shared persistence mechanisms [63, 64]. Colonization of both CRKP and ESBL isolates could be attributed not only to resistance genes but also to enhanced persistence pathway within the host gut and potentially surrounding environment [65].

Genetic analysis showed the crucial explanation for these phenotypic differences. The resistance in the CRKP group was mainly associated with the presence of carbapenemase genes, specifically *bla*_NDM_ (54.5%) and *bla*_KPC_ (45.5%), both of which were completely absent in the ESBL group. *Bla*_*NDM*_ and *bla*_*KPC*_ enzymes have the ability to hydrolyze carbapenems, which limit the therapeutic value of these essential class of antibiotics. This contrasts with ESBLs, which primarily affect narrower-spectrum β-lactams but spare carbapenems [66]. The coexistence of these genes, along with the high prevalence of *bla*_CTX−M−1_ (86.4%), indicates that the CRKP isolates are poly-resistant and harbor a complex array of resistance determinants, making treatment options extremely limited. The co-carriage of NDM and KPC genes is a particularly serious combination, often associated with highly transferable mobile genetic elements, facilitating the rapid spread of resistance. Many clinical settings from Egypt, reported the simultaneous existence of *bla*_NDM_ and *bla*_KPC_ genes within *K. pneumoniae* isolate. A genomic study from two tertiary care hospitals, identified high-risk clones as ST11 that co-harbor *bla*_NDM−1_ and *bla*_KPC−2_. Within the Egyptian context, several studies have documented varied rates of individual carbapenemase gene carriage; one Egyptian survey found *bla*_KPC_ in approximately 4% and *bla*_NDM_ in 3.8% of isolates, while other reports from neonatal ICU showed that *bla*_KPC_ was detected in up to 95.8% of extensively drug-resistant *K*. *pneumoniae* neonatal isolates, all of which carried *bla*_NDM_ [67, 68]. Moreover, this coexistence of carbapenemase phenotype has drawn global attention. In the Middle East and South Asia, CRKP revealed a 57.8% increase across surveyed countries between 2019 and 2023, with *bla*_NDM_ prevalence climbing dramatically —from 7.8% to 59.3% over a six-year period in India alone — alongside co-occurrence of multiple resistance genes becoming increasingly common [69, 70]. The global spread of dual-enzyme lineages, their enrichment in ICU environments, and their association with pan-drug resistance make them among the most formidable infectious threats in contemporary hospital medicine [71, 72].

This contrasts with ESBLs, which primarily affect narrower-spectrum β-lactams but spare carbapenems [66]. The ESBL phenotype, in contrast, is associated with *bla*_CTX−M_, *bla*_TEM_, and *bla*_SHV_ genes, which are common worldwide [73–75].

Antibiotic usage record showed that the main therapeutic strategy in the current study included dual β-lactam combinations, most commonly combinations involved Ampicillin + Sulbactam + Cefepime and Ampicillin + Sulbactam + Ceftazidime. The excessive use of broad spectrum β-lactams combination during the current study represent a selective pressure induce the carriage of both ESBL and CRKP strains. Reports during the COVID-19 pandemic revealed higher ESBL-producing Enterobacterales (ESBL-PE) incidence in severe COVID-19 patients, that was attributed to use of broad-spectrum β-lactam. One ICU study observed that use of cefotaxime was the main factor for ESBL-PE acquisition in severe COVID-19 patients. Moreover, Italian studies included 1617 patients descried high ESBL-PE, mainly Kp-ESBL, in COVID-19 wards compared to other wards [76]. The incidence of CRKP isolation rose 4.8 times during the COVID-19 era, particularly in intensive care units where patients were ventilated and required broad-spectrum antimicrobials for associated complications. In about 50% of CRKP cases, a coexistence with COVID-19 infection was observed. It is commonly involving a complication such as secondary pneumonia. These conditions likely contributed to selective antibiotic pressures from β-lactams such as cephalosporins and carbapenems. The abuse of these antimicrobials during the pandemic was associated with resistance shifts, including from ESBL to carbapenemase production [77]. Rational antimicrobial use, new agents such as cefiderocol, and infection controls would reduce the spread of resistant pathogens. The increased resistance levels to cephalosporins in these patients reflect their inadequacy against the circulating strains as an empirical choice. Our study identified exposure to antibiotics as a risk factor, but the cause-and-effect relationship of a particular antibiotic regimen leading to colonization needs further analysis using prospective designs.

The cross-sectional, single-center design and small sample size of this study hinder generalizability and causal inference. The absence of whole-genome sequencing due to resource constraints limited our ability to evaluate clonal connections and plasmid-mediated resistance transmission. Another limitation of our report is that fecal samples were collected after hospitalization and antibiotic exposure. Consequently, it is quite unclear whether colonization was community acquired or it was acquired during hospitalization period. Future multicenter, prospective studies with repeated sampling, thorough antimicrobial data, and genomic characterization are required to elucidate colonization dynamics, resistance transmission, and clinical impact.

## Conclusion

The current investigation indicated a high prevalence of fecal colonization by MDR *K. pneumoniae* among hospitalized pediatric patients with COVID-19. CRKP represented the predominant resistant phenotype. CRKP isolates showed a pronounced propensity for biofilm production, a high prevalence of the carbapenemase-encoding genes *bla*_NDM_ and *bla*_KPC_, and extensive resistance to multiple antimicrobial classes. Our findings highlight the coexistence of antimicrobial resistance and persistence strategies within the intestinal microbiota. This underlines the role of gut colonization as an essential reservoir for antimicrobial resistance development and facilitating nosocomial dissemination among vulnerable patients. The use of broad-spectrum antimicrobial therapies observed in this cohort underscores the necessity for improving antimicrobial stewardship, conducting active surveillance, implementing strict infection prevention and control measures. Routine monitoring of fecal carriage may provide valuable epidemiological insights for identifying patients at increased risk of subsequent infection and hospital-associated transmission.

## Supplementary Information

Supplementary Material 1.

## Data Availability

The authors declare the availability of the data when required on reasonable request.
