# Supplementary material for: Molecular and phenotypic profiles of carbapenem- and third-generation cephalosporin-resistant Klebsiella pneumoniae isolates from the fecal microbiota of pediatric patients with COVID-19
Source: BMC Microbiol. 2026 Jul 23;26:664. doi: 10.1186/s12866-026-05426-5 (PMC13393905; doi:10.1186/s12866-026-05426-5)

**Supplementary Data:**

**Table S1: Primer list for detection of β-lactamase and carbapenemase genes.**

| Target gene | Primer name | Primer sequence (5′–3′) | Product size (bp) | PCR type | Reference |
| --- | --- | --- | --- | --- | --- |
| blaTEM | TEM-F | ATGAGTATTCAACATTTCCG | 800 | Multiplex | Dallenne et al., 2010 |
|  | TEM-R | CTGACAGTTACCAATGCTTA |  |  |  |
| blaSHV | SHV-F | ATGCGTTATATTCGCCTGTG | 713 | Multiplex | Dallenne et al., 2010 |
|  | SHV-R | TGCTTTGTTATTCGGGCCAA |  |  |  |
| blaOXA-1-like | OXA-1-F | ATGAAAAACACAATACATATCAACTTCGC | 564 | Multiplex | Dallenne et al., 2010 |
|  | OXA-1-R | GTGTGTTTAGAATGGTGATCGCATT |  |  |  |
| blaCTX-M group 1 | CTX-M-1-F | SCSATGTGCAGYACCAGTAA | 688 | Multiplex | Dallenne et al., 2010 |
|  | CTX-M-1-R | CCGCRATATGRTTGGTGGTG |  |  |  |
| blaCTX-M group 2 | CTX-M-2-F | CGACGCTACCCCTGCTATT | 404 | Multiplex | Dallenne et al., 2010 |
|  | CTX-M-2-R | CCAGCGTCAGATTTTTCAGG |  |  |  |
| blaCTX-M group 9 | CTX-M-9-F | CAAAGAGAGTGCAACGGATG | 561 | Multiplex | Dallenne et al., 2010 |
|  | CTX-M-9-R | ATTGGAAAGCGTTCATCACC |  |  |  |
| blaCTX-M-8/25 | CTX-M-8/25-F | AACRAGCGGCGTTGCTT | 666 | Uniplex | Dallenne et al., 2010 |
|  | CTX-M-8/25-R | TCGGRTTCAGTTTTGCC |  |  |  |
| blaKPC | KPC-F | ATGTCACTGTATCGCCGTCT | 798 | Uniplex | Chen et al., 2011 |
|  | KPC-R | TTTTCAGAGCCTTACTGCCC |  |  |  |
| blaVIM | VIM-F | GATGGTGTTTGGTCGCATA | 390 | Uniplex | Poirel et al., 2011 |
|  | VIM-R | CGAATGCGCAGCACCAG |  |  |  |
| blaIMP | IMP-F | GGAATAGAGTGGCTTAAYTCTC | 232 | Uniplex | Poirel et al., 2011 |
|  | IMP-R | GGTTTAAYAAAACAACCACC |  |  |  |
| blaNDM | NDM-F | GGTTTGGCGATCTGGTTTTC | 621 | Uniplex | Poirel et al., 2011 |
|  | NDM-R | CGGAATGGCTCATCACGATC |  |  |  |

Description of primer pairs employed in PCR reactions for the detection of antibiotic resistance genes, such as β-lactamases (ESBLs) [blaTEM, blaSHV, and blaCTX-M families], and carbapenemase genes [blaKPC, blaVIM, blaIMP, and blaNDM]. Sequences of primers are mentioned below in the 5'-to-3' direction. Expected sizes of the amplified products, type of PCR

**Table S2: Prevalence of antimicrobial resistance genes among CRKP and ESBL-producing isolates.**

| Gene | CRKP (n=22) | ESBL (n=14) | p-value | OR (95% CI) |
| --- | --- | --- | --- | --- |
| CTXM-1 | 19 (86.4%) | 8 (57.1%) | 0.08 | 4.75 (0.97-23.3) |
| CTXM-2 | 6 (27.3%) | 4 (28.6%) | 0.94 | 0.94 (0.21-4.15) |
| CTXM-9 | 3 (13.6%) | 4 (28.6%) | 0.38 | 0.40 (0.07-2.14) |
| TEM | 11 (50.0%) | 7 (50.0%) | 1.00 | 1.00 (0.28-3.59) |
| SHV | 12 (54.5%) | 5 (35.7%) | 0.49 | 2.16 (0.43-6.56) |
| OXA-1 | 4 (18.2%) | 3 (21.4%) | 0.82 | 0.82 (0.15-4.34) |
| KPC | 10 (45.5%) | 0 (0%) | <0.0001 | ∞ |
| NDM | 12 (54.5%) | 0 (0%) | <0.0001 | ∞ |
| IMP-2 | 0 (0%) | 0 (0%) | - | - |
| VIM | 0 (0%) | 0 (0%) | - | - |

Relationships between groups were determined by calculating the odds ratio (OR) with 95% confidence interval (CI) and respective p-value. Infinite OR value suggests that the presence of genes was unique to the CRKP group. There was no blaIMP-2 and blaVIM in any isolate

**Table S3: Frequency of antimicrobial regimens received by patients infected or colonized with CRKP and ESBL-producing organisms during the hospitalization period.**

| Combination | CRKP Count | ESBL Count | Total |
| --- | --- | --- | --- |
| "Ampicillin + Sulbactam + Cefepime" | 7 | 6 | 13 |
| "Ampicillin + Sulbactam + Ceftazidime" | 7 | 4 | 11 |
| "Vancomycin + Ceftriaxone" | 2 | 0 | 2 |
| "Vancomycin + Cefotaxime " | 0 | 1 | 1 |
| "Ampicillin + Sulbactam + Cefotaxime" | 2 | 0 | 2 |
| "Ampicillin + Sulbactam + Ceftazidime + Metronidazole" | 1 | 0 | 1 |
| "Ampicillin + Sulbactam + Cefotaxime + Azithromycin" | 1 | 1 | 2 |
| "Meropenem + Acyclovir" | 1 | 0 | 1 |
| "Vancomycin + Meropenem" | 1 | 0 | 1 |
| "Clindamycin + Cefepime + Linezolid" | 0 | 2 | 2 |

Counts represent the number of patients receiving each antibiotic combination. The total column indicates the combined frequency across both study groups. Multiple antibiotic combinations may have been administered according to clinical management and disease severity.

**Figure S1: Triton Hodge Test Demonstrating Carbapenemase Production in *K. pneumoniae* Isolates.** **Triton** **Hodge test (THT) showing carbapenemase activity among *K. pneumoniae* isolates. The central meropenem (MRP 10 µg) disk is surrounded by streaks of test isolates (R1, R3, R4, and R7). The characteristic cloverleaf-like indentation of bacterial growth toward the carbapenem disk indicates positive carbapenemase production.**


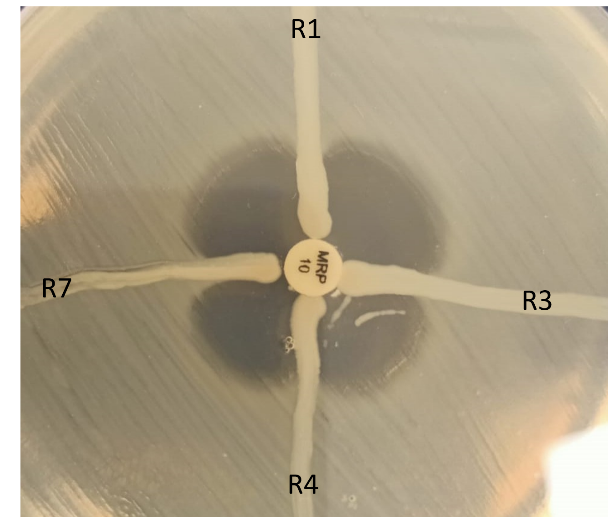

Supplement: Supplementary file 1 — Supplementary Material 1. [file 12866_2026_5426_MOESM1_ESM.docx]
